# Extending protein interaction networks using proteoforms and small molecules

**DOI:** 10.1093/bioinformatics/btad598

**Published:** 2023-09-26

**Authors:** Luis Francisco Hernández Sánchez, Bram Burger, Rodrigo Alexander Castro Campos, Stefan Johansson, Pål Rasmus Njølstad, Harald Barsnes, Marc Vaudel

**Affiliations:** Department of Clinical Science, Mohn Center for Diabetes Precision Medicine, University of Bergen, Bergen 5020, Norway; Department of Medical Genetics, Center for Medical Genetics and Molecular Medicine, Haukeland University Hospital, Bergen 5020, Norway; Department of Clinical Science, Mohn Center for Diabetes Precision Medicine, University of Bergen, Bergen 5020, Norway; Department of Medical Genetics, Center for Medical Genetics and Molecular Medicine, Haukeland University Hospital, Bergen 5020, Norway; Department of Biomedicine, Proteomics Unit, University of Bergen, Bergen 5020, Norway; Department of Informatics, Computational Biology Unit, University of Bergen, Bergen 5020, Norway; Departamento de Sistemas, Universidad Autónoma Metropolitana Azcapotzalco, Mexico City 02128, Mexico; Department of Clinical Science, Mohn Center for Diabetes Precision Medicine, University of Bergen, Bergen 5020, Norway; Department of Medical Genetics, Center for Medical Genetics and Molecular Medicine, Haukeland University Hospital, Bergen 5020, Norway; Department of Clinical Science, Mohn Center for Diabetes Precision Medicine, University of Bergen, Bergen 5020, Norway; Department of Pediatrics, Haukeland University Hospital, Bergen 5020, Norway; Department of Biomedicine, Proteomics Unit, University of Bergen, Bergen 5020, Norway; Department of Informatics, Computational Biology Unit, University of Bergen, Bergen 5020, Norway; Department of Clinical Science, Mohn Center for Diabetes Precision Medicine, University of Bergen, Bergen 5020, Norway; Department of Informatics, Computational Biology Unit, University of Bergen, Bergen 5020, Norway; Department of Genetics and Bioinformatics, Health Data and Digitalization, Norwegian Institute of Public Health, Oslo 0213, Norway

## Abstract

**Motivation:**

Biological network analysis for high-throughput biomedical data interpretation relies heavily on topological characteristics. Networks are commonly composed of nodes representing genes or proteins that are connected by edges when interacting. In this study, we use the rich information available in the Reactome pathway database to build biological networks accounting for small molecules and proteoforms modeled using protein isoforms and post-translational modifications to study the topological changes induced by this refinement of the network representation.

**Results:**

We find that improving the interactome modeling increases the number of nodes and interactions, but that isoform and post-translational modification annotation is still limited compared to what can be expected biologically. We also note that small molecule information can distort the topology of the network due to the high connectedness of these molecules, which does not necessarily represent the reality of biology. However, by restricting the connections of small molecules to the context of biochemical reactions, we find that these improve the overall connectedness of the network and reduce the prevalence of isolated components and nodes. Overall, changing the representation of the network alters the prevalence of articulation points and bridges globally but also within and across pathways. Hence, some molecules can gain or lose in biological importance depending on the level of detail of the representation of the biological system, which might in turn impact network-based studies of diseases or druggability.

**Availability and implementation:**

Networks are constructed based on data publicly available in the Reactome Pathway knowledgebase: reactome.org.

## 1 Introduction

Biological networks are a promising way to interpret modern biomedical data at scale (Sonawane *et al.* 2019). They allow the study of molecular patterns at both local and global scale, and hence provide a systemic view on molecular processes. The fundamental building blocks of a biological network are the interactions between biological entities, with the entities themselves represented by nodes and their interactions by connections (Burger *et al.* 2018). The entire collection of interactions in a biological system is called the interactome. Interactions between biomolecules leading to biochemical transformations are grouped into reactions, and combinations of reactions achieving a biological function are termed pathways. The main participants of pathways are proteins, which can interact with themselves, other proteins, or other molecules. It is common in the study of biological networks to focus on protein–protein interactions and to represent proteins by the name of the genes encoding them. Genes form a more generic network model that lacks molecular details of the proteins, but that captures enough information to draw meaningful inference on the interactome (Menche *et al.* 2015).

A relationship between nodes of the network can be inferred from multiple sources: text mining, co-expression, physical interaction, or from literature knowledge on the functions of proteins (Fernández-Tajes *et al.* 2019). Such networks have proved to be particularly useful for understanding biological mechanisms (Huttlin *et al.* 2017, Dimitrakopoulos *et al.* 2018, Reyna *et al.* 2020). For example, gene network approaches have been used for analyzing functions of genes associated with different types of cancer (Wu and Stein 2012, Creixell *et al.* 2015). Based on a given interactome, network analyses attempt to extract knowledge concerning specific sets of proteins. For example, *guilty by association* procedures assume that proteins colocalizing in the network are functionally related (Creixell *et al.* 2015). Similarly, diffusion models estimate the effects of gene alterations towards their neighborhood (Vandin *et al.* 2011). By design, such network analysis methods rely heavily on network structural properties such as the number of neighbors per node or the number of connections between groups of nodes (Sonawane *et al.*, 2019). It is then vital to carefully choose what the nodes and connections represent, such that any inference from the network mirrors the reality of biological systems.

In practice, as a result of genetic variation, RNA splicing, and post-translational modification (PTM), a gene can yield many distinct forms of a protein, called proteoforms (Smith *et al.* 2013). For most proteins, the different isoforms of a gene share <50% of interactions (Yang *et al.* 2016). For example, *Bcl-2* has two isoform products Bcl-xl and Bcl-xs resulting from alternative splicing. Bcl-xl, which contains the BH1 and BH2 domains, is responsible for programmed cell death, while Bcl-xs lacks both domains, therefore contributing to the opposite function (Schwerk and Schulze-Osthoff 2005). One can legitimately anticipate an even higher specificity when including PTMs. However, this information is lost when creating biological networks using gene names as sole descriptor of the protein. Another source of information lost in the construction of gene-centric networks is the role of molecules that are not encoded in the genome and would therefore not be represented in a gene-network. Such molecules are referred to as *SimpleEntity* in the Reactome object model and are called small molecules throughout this study. Small molecules play essential roles in biological systems, e.g. they include metabolites participating as reactants, catalyzers, or inhibitors of reactions. For example, adenosine triphosphate (ATP) and guanosine triphosphate (GTP) are essential metabolites needed as energy sources. ATP hydrolysis provides the energy for protein transport in the mitochondria, for binding and releasing the newly synthesized polypeptide molecules from the *hsp70* chaperone proteins (Jinek *et al.* 2020).

Previously, we have demonstrated that it is possible to leverage the rich information contained in the Reactome pathway knowledgebase to refine the representation of biological networks by accounting for proteoform-specificity of biological reactions (Sánchez *et al.* 2019). Here, we demonstrate how changing the type of node from gene to proteoform influences the structure of the obtained networks. In addition, we study how the inclusion of small molecules affects the representation of the network. Together, our results show that changing the representation of biological networks can help refine the modeling of biological processes, but that the limited information on proteoform-specific interactions still impairs the application of such approaches at scale.

## 2 Materials and methods

### 2.1 Reactome data

Reference knowledge to conduct the analysis was obtained from the Reactome graph database (version 84). The database dump file (reactome.org/download-data) was loaded and run using Neo4j Desktop 1.5.7 to Neo4j Graph Database Manager 4.4.19. The analysis scripts were implemented using Python 3.11 organized as Jupyter notebooks. They communicated with the database management system using the Neo4j Python Driver Manual version 5.7.0.

### 2.2 Network construction

The Reactome graph database data model is organized as nodes and relationships with properties and labels. We used *Event* entities to infer the nodes and connections for the network. Events involve the transformation of input nodes into output nodes in one or multiple steps. We queried for two types of event nodes: *Pathway* and *ReactionLikeEvents*. *ReactionLikeEvents* convert input entities to output entities in one step, while *Pathways* group sets of *ReactionsLikeEvents*. Each event has participant molecules which perform roles of input (reactant), output (product), regulators and catalyzers (enzymes). The data model represents events occurring in sequence by annotating the output of the first event as input of the second event.

Participants of reactions are *physical entities*, which are of two main types: *accessioned entities* and *simple entities*. Accessioned entities stand for molecules that feature a standard identifier for each sequence pattern, typically nucleotide-based sequences (genes or transcripts) or amino acid-based sequences (proteins). They are annotated with references to the genes possibly encoding them, annotated with HUGO gene nomenclature identifiers (Tweedie *et al.* 2021), while proteins have UniProt (UniProt Consortium 2020) accession numbers. Simple entities, referred to as small molecules throughout this work, refer to fully characterized molecules that are not genome encoded. They include metabolites but are not restricted to these. Small molecules have unique identifiers from the Chemical Entities of Biological Interest (ChEBI) database (Hastings *et al.* 2016). The other, rarer, types of participants include drugs, polymers, and those labeled *OtherEntity* in Reactome, and were not included in this study.

When this information is available, accessioned sequence participants are additionally annotated with their isoform and the minimal set of post-translational modifications necessary to perform their role in the biological event. Combining the set of modifications and isoform sequence, we built a theoretical proteoform state in which the gene products need to be present to participate in a given reaction.

Participants of events may also be *entity sets*, *complexes*, and *genome encoded* entities. Entity sets stand for groups of entities which may be used almost interchangeably within a biological event. For example, multiple proteins may interchangeably perform the same role in a reaction, e.g. catalyzing a reaction. Complexes are the conjunction of multiple molecules into a single unit. The members of the complex may be of all other types of participants, i.e. accessioned sequences, simple entities, or even complexes. Genome encoded entities are protein or nucleic acid molecules whose sequence is not yet clearly defined for a specific species.

We constructed gene- and proteoform-centric interaction network representations of the Pathways in Reactome by taking all reaction participants as nodes of the network, as in previous studies (Burger *et al.* 2018, Sánchez *et al.* 2019). We considered all entities with gene, protein, or chemical accessions. We did not consider genome encoded entities that did not have a reference identifier and did not include molecules without a chemical accession.

For the gene-centric representation, all physical entities associated with a given gene and each of its associated UniProt protein accessions are represented by a single node, i.e. merging all protein products, isoforms, and proteoforms into one node. For the proteoform-centric representation, we represented each proteoform with a separate node. We took the associated protein accession, the isoform, and the set of post-translational modifications annotated to represent a single proteoform. Then, all physical entities yielding the same isoform with the same sequence modification combinations were represented by a single node. For both the gene- and proteoform-centric networks we constructed two alternative networks that additionally considered the simple entities, i.e. small molecules in this study; the first alternative adds a single node for each small molecule, the second alternative adds a node for each small molecule to every reaction in which the given small molecule participates. We included all participant accessioned entities and small molecules directly as nodes, splitting complexes and entity sets into the single accessioned entities and small molecules they consist of.

Once the nodes were defined, we created a connection between each pair of nodes that was annotated to perform a role in the same reaction, such as input and output. To construct a complete interactome we processed all pathways with all their respective reactions to obtain their nodes and connections. Nodes were not repeated, but instead we aggregated their connections obtained from the different pathways. All connections were undirected.

### 2.3 Pathway-specific subnetworks

To build subnetworks representing a single pathway, we listed the reactions the given pathway consists of, and built connections and nodes like done for the entire network. If a pathway was found to contain another pathway, the subnetwork of the subpathway was constructed and merged with the network of the pathway.

### 2.4 Network analyses

Networks were represented and analyzed using the Networkx library version 3.1 for Python. The library allows the calculation of size, articulation points, bridges, and connected components.

Addition, we implemented our own procedure for percolation analysis: 10% of the connections from the network were randomly removed, and the size of the largest connected component (LCC), also called *giant component*, was monitored; this procedure was repeated iteratively until no connection remained. The average size of the LCC was plotted against the share of nodes remaining in the LCC, called network completeness. The point where the size of the LCC collapses rapidly represents the percolation threshold, a topological metric of the network (Menche *et al.* 2015). For the creation of Fig. 3, the procedure was conducted twenty times for every network; the dots represent a point in the percolation and the solid line the average LCC size at a given completeness.

### 2.5 Code availability

All the code used to construct the networks and replicate the topological analysis is publicly available at the public repository: github.com/PathwayAnalysisPlatform/ProteoformNetworks.

### 2.6 Data availability

All the data used in this study are available at the Reactome website: reactome.org.

The networks produced by this study are available at the public repository: github.com/PathwayAnalysisPlatform/Networks.

## 3 Results

### 3.1 Increased size of the interactome

A recent estimate for the human genome lists approximately 47 000 genes, of which approximately 19 000 are coding for proteins (Tweedie *et al.* 2021). The estimated number of protein products resulting from alternative splicing is around 70 000 isoforms (Aken *et al.* 2017). The total number of functional proteoforms remains unknown, but estimates are in the millions depending on how proteoforms are defined (Aebersold *et al.* 2018). Changing the representation of a network from a gene-centric to a proteoform-centric paradigm should therefore result in a network several orders of magnitude larger. Based on isoform and post-translational modification information from the Reactome knowledgebase v84 for *Homo Sapiens*, we can represent 14 253 distinct proteoforms participating in 14 212 reactions (see Section 2 for details). These 14 253 proteoforms represent 11 057 proteins linking to 11 039 gene names, making 1.29 proteoforms per gene on average. We constructed a network based on all pathways in Reactome by connecting entities when they participate in the same reaction. Building the network based on proteoforms instead of genes yields 3214 (+29.1%) additional nodes and 216 204 (+58.6%) additional connections. Thus, while the proteoform annotation provides enough information to substantially increase the size of the network, out of the millions of expected proteoforms, only a few proteoforms are annotated functionally.

The genes which encode the proteins with the highest number of proteoforms annotated are *UBC* (Polyubiquitin-C), *H3C1* (Histone H3.1), and *H3C15* (Histone H3.2), with 55, 52, and 48 proteoforms, respectively, participating in diverse pathways and located in multiple subcellular compartments (Supplementary Table S1). *UBC*, e.g. has products mostly ubiquitinylated or with crosslinks between l-lysine residues and glycine at multiple locations of the sequence, generating a high number of proteoforms representing different combinations of post-translational modifications. For these examples, the proteoform representation of biological interactions was completely different compared to a gene-centric network. Another factor that can influence the respective number of proteoforms per gene is the ambiguity of identifier mapping between databases, where one gene can map to multiple proteins, and the other way around, although the prevalence of this problem diminishes as identifier mapping gets harmonized between resources.

Reactome also contains small molecules annotated as participants of human reactions. Extending the gene- and proteoform-centric networks with small molecules increases the number of nodes by 2070, representing an increase of 18.8% and 14.5%, respectively (Table 1). Adding small molecules creates 86 195 and 91 921 new connections, corresponding to an increase of 23.3% and 15.7% for the gene- and proteoform-centric networks, respectively. However, this creates situations where small molecules ubiquitous in biochemical reactions, like H_2_O or ATP, connect most of the network. To take the influence of small molecules into account without distorting the network globally, we introduced the possibility for small molecules to connect pathway participants within but not between reactions. The number of new connections then becomes 446 415 and 456 820, corresponding to an increase of 120.9% and 78.0%, for the gene- and proteoform-centric networks, respectively.

**Table 1.** Sizes of the six alternative interactome networks resulting from combining entity level (genes, proteoforms) and three options to consider small molecule nodes.^a^

Small molecules	Entity level	Interactions	Nodes	Num accessioned entities	Num small molecules
Not included	Genes	369 224	11 039	11 039	0
Not included	Proteoforms	585 428	14 253	14 253	0
Included	Genes	455 419	13v109	11 039	2070
Included	Proteoforms	677 349	16 323	14 253	2070
Reaction-unique included	Genes	815 639	40 949	11 039	29 910
Reaction-unique included	Proteoforms	1 042 248	44 163	14 253	29 910

a Sizes are shown as number of connections (interactions) and number of nodes.

### 3.2 Interconnected proteoforms alter the degree distribution

**Figure 1.**
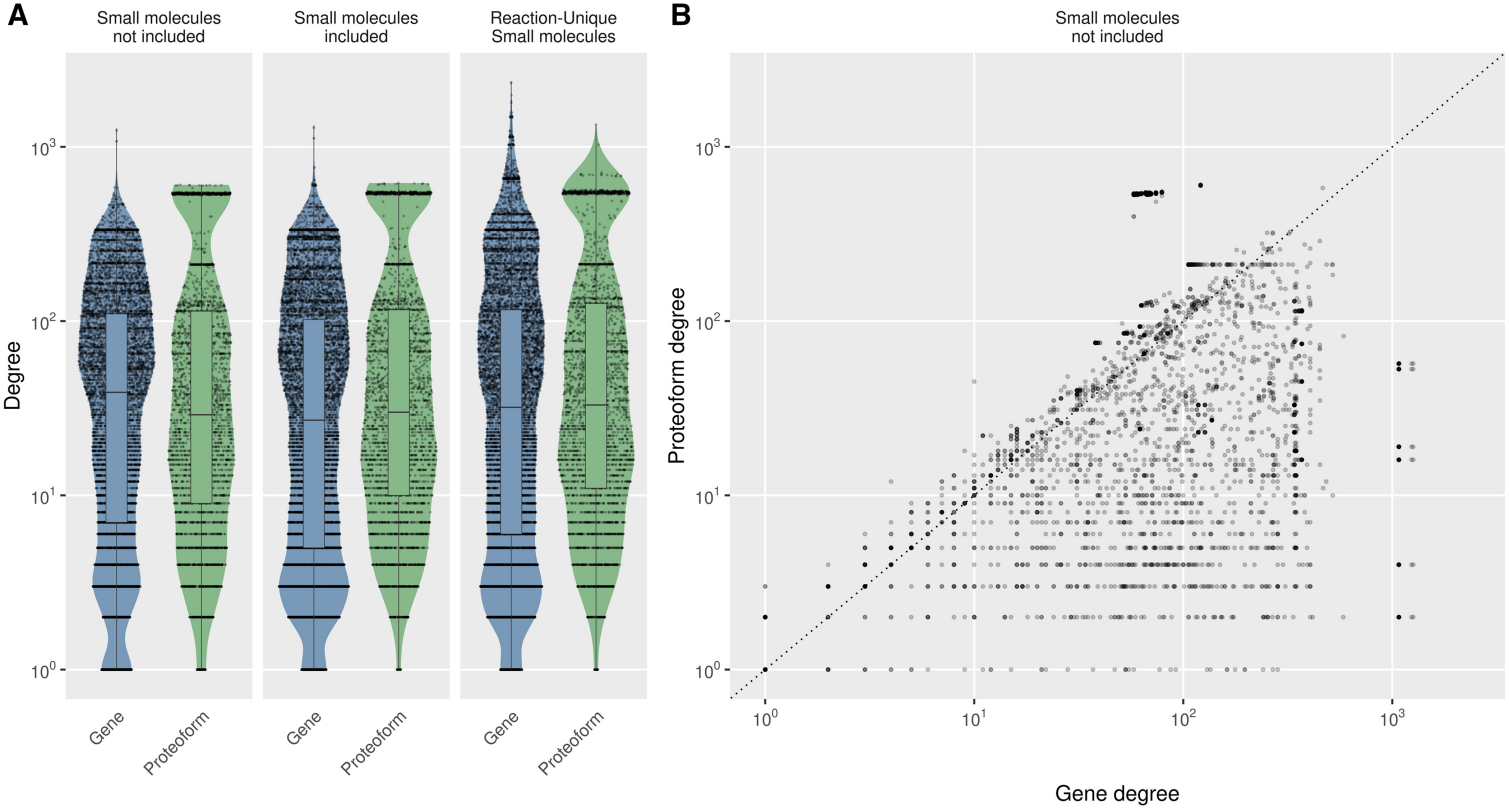
Node degree distribution for nodes with isoform or post-translational modifications annotated in the different interactomes depending on how small molecules are considered and whether proteoforms are considered as individual nodes or collapsed into a single gene. (A) Left: only gene or proteoform nodes; no small molecule nodes. Center: small molecule nodes included in the network; one node for each. Right: “reaction-unique” small molecule nodes included; adding one separate node for each reaction where the small molecule participates. (B) Degree values for each node for interactomes without small molecules. Comparison of gene node degree versus proteoform node degrees. Includes only proteoforms with isoform or post-translational annotations. Each node is plotted with a transparent dark point. A dotted line shows where the degree is the same for both types of nodes.

The connectivity of a node in a network is measured by the number of connections, also called the degree. Without accounting for small molecules, 143 295 (24.5%) of the connections in the proteoform-centric network represent connections where proteoform-level information is available for both nodes, while 101 746 (17.4%) and 340 387 (58.1%) of the connections present no proteoform annotation for one or both nodes, respectively. Since proteoforms are specific forms of a protein (Smith *et al.* 2013), changing a gene-centric network into a proteoform-centric representation can be seen as distributing the protein–protein interactions between new, more specific, nodes. Thus, intuitively, proteoform nodes are expected to have a smaller degree than the gene that encodes them. As we previously described (Sánchez *et al.* 2019), the majority of proteoforms (57.1%) indeed present a degree lower than their genes in the gene-centric network, but a minority (35.4%) increases its degree due to proteoform-proteoform interactions. The degree distributions for nodes annotated with isoform or PTMs are plotted in Fig. 1 and Supplementary Fig. S1, and summary statistics on the overall degree in the networks are detailed in Supplementary Table S2.

This is, e.g. the case for collagen-related genes such as *COL7A1*, *COL3A1*, and *COL6A3*, which present much higher degrees in the proteoform-centric than in the gene-centric network: 606 versus 121, 547 versus 67, and 546 versus 66, respectively. These collagen nodes are expanded to a wide variety of proteoforms as they become multiply modified by sequential reactions. For example, in the pathway *Collagen biosynthesis* a reaction converts *collagen lysines* to *5-hydroxylysines*, and diverse *COL7A1* gene products are input and output of the reaction. In a gene-centric network, this reaction is modeled as a single *COL7A1* gene node, while in the proteoform-centric network, the input nodes *COL7A1, 3x4Hyp-COL7A1*, and *3x4Hyp-3Hyp-COL7A1* are connected to the output nodes *5Hyl-COL7A1, 3x4Hyp-5Hyl-COL7A1*, and *3x4Hyp-3Hyp-5Hyl-COL7A1*, yielding nodes with higher detail of information but also with higher degree than in the gene-centric network. Other nodes that consequently have their degree increased do not necessarily have proteoform-level annotation, such as *PLOD3*, which has its degree increased by an order of magnitude (from 46 to 529), simply because it participates in reactions with multiple collagen gene products, therefore connecting to many proteoforms. The node degrees for gene- and proteoform-centric networks are listed in Supplementary Table S3.

As detailed in Supplementary Table S2, when extending the networks with small molecules, the average degree of accessioned entity nodes, i.e. genes or proteoforms, increased from 66.9 to 73.1 (+9.3%) and from 82.2 to 87.4 (+6.3%) for genes and proteoforms, respectively. For the majority of nodes, the degree of small molecules is lower than the degree of accessioned entities, the resulting median degree in the overall network is therefore lower or very similar (Fig. 1). As previously introduced, the ubiquitousness of small molecules however produces hyperconnected nodes with up to 3643 and 4308 connections in the gene-centric and proteoform-centric networks, respectively, while the most connected genes and proteoforms present 1306 and 1552 connections, respectively. Restricting small molecules to reaction-specific relationships allows considering the local function of small molecules without creating hyperconnected small molecule nodes, decreasing their average degree from 50.2 to 17.8 and from 53.0 to 19.1 for the gene and proteoform networks, respectively. The average degree of accessioned entity nodes is further increased to 99.5 and to 108.2 for the gene- and the proteoform-centric networks, respectively, while the maximal degree increases to 2349 and 2364, and the maximal degree of small molecules remains 304 in both networks. The increase in maximal degree when compartmenting small molecules per reaction can be explained by highly connected genes or proteoforms that participate in many reactions with the same small molecule: when building reaction-specific small molecules the degree of the gene or proteoform increases by the number of reactions.

While interactome-wide studies have proven valuable in biomedicine, they lack biological context, e.g. in time and space, and can thus yield results that are biologically unlikely or impossible: it is not because A can interact with B and B with C, that the path A, B, C is possible. We used the pathways in Reactome to build bona fide subnetworks of chained reactions and study the influence of introducing proteoforms on these subnetworks, thereby providing a local view complementary to the global results obtained on the entire interactome. We focused on the pathways that contain at least one node with isoform or modification information (1134 pathways from a total of 2138). Note that pathways are organized in a hierarchical structure and we included all pathways, i.e. also pathways containing other pathways, some nodes and reactions are therefore redundant between pathways (Burger *et al.* 2018). The sizes of the pathway-based subnetworks are displayed in Supplementary Fig. S2. The average degree per proteoform, 17.35, was slightly higher than per gene, 15.5 (+11.9%) (Supplementary Table S4). It therefore appears that the increase in degree observed for the whole network is preserved for within-pathway connections, and not only due to between-pathway connections between proteoforms and other proteins.

### 3.3 Proteoforms modify the properties of connected components

**Figure 2.**
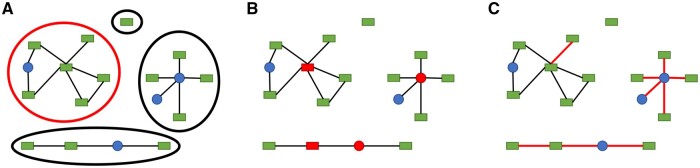
Illustration of graph theory concepts using hypothetical networks with proteoform nodes (green rectangles) and small molecule nodes (blue circles). (A) Connected components of the network, each one surrounded with a dotted line. Largest connected component highlighted with red dotted line. (B) Articulation points, nodes highlighted in red. (C) Bridges, connections highlighted with red lines.

*Connected components* are the maximal subnetworks in which all nodes of the component can reach each other through a path (Fig. 2). A subnetwork is a network containing a subset of nodes or connections from the original network. The Largest Connected Component (LCC) of a network is the component with the highest number of nodes. In our analysis of Reactome, gene- and proteoform-centric networks showed similar relative size of the LCC (Supplementary Table S5), both globally and per pathway (Supplementary Table S6).

The proteoform interactome extends gene nodes into multiple proteoform nodes. Proteoforms resulting from variation of a single gene, called a proteoform family, may participate in disjoint sets of reactions in the network. If gene nodes are represented by multiple proteoforms participating in separate reactions or pathways, the overlap will only be observable at the gene level and not at the proteoform level. In other words, proteoforms from a single gene may be split over different subnetworks, e.g. pathways or nodes of interest, and even different connected components. In this case, subnetworks would intersect in the gene-centric representation of the network, but not in the proteoform-centric representation, where the different subnetworks would be disconnected.

We found 505 proteins where at least one proteoform of the family participates in a biochemical reaction where the other members of the family are not involved. Identifying such a proteoform in a sample therefore provides pathway-specific information that is lost in a gene-centric representation, as in that case all reactions and pathways where any of the family members participate become indistinguishable. As an example, the human protein Peroxiredoxin-5 (P30044) has isoforms P30044-1 located at the Mitochondrial Matrix, and P30044-2 in the Cytosol. They differ in sequence, the second one missing the first 52 amino acids, and participate in separate reactions in different subcellular locations: “*PRDX5 reduces peroxynitrite to nitrite using TXN2*” and “*PRDX1,2,5 catalyzes TXN reduced + H2O2 => TXN oxidized + 2H2O*”, respectively. In this case, a proteoform-centric representation would distinguish the mitochondrial from the cytosol reaction, connecting them through the translocation and processing of P30044 into P30044-1, while a gene-centric representation would make both reactions indistinguishable.

**Figure 3.**
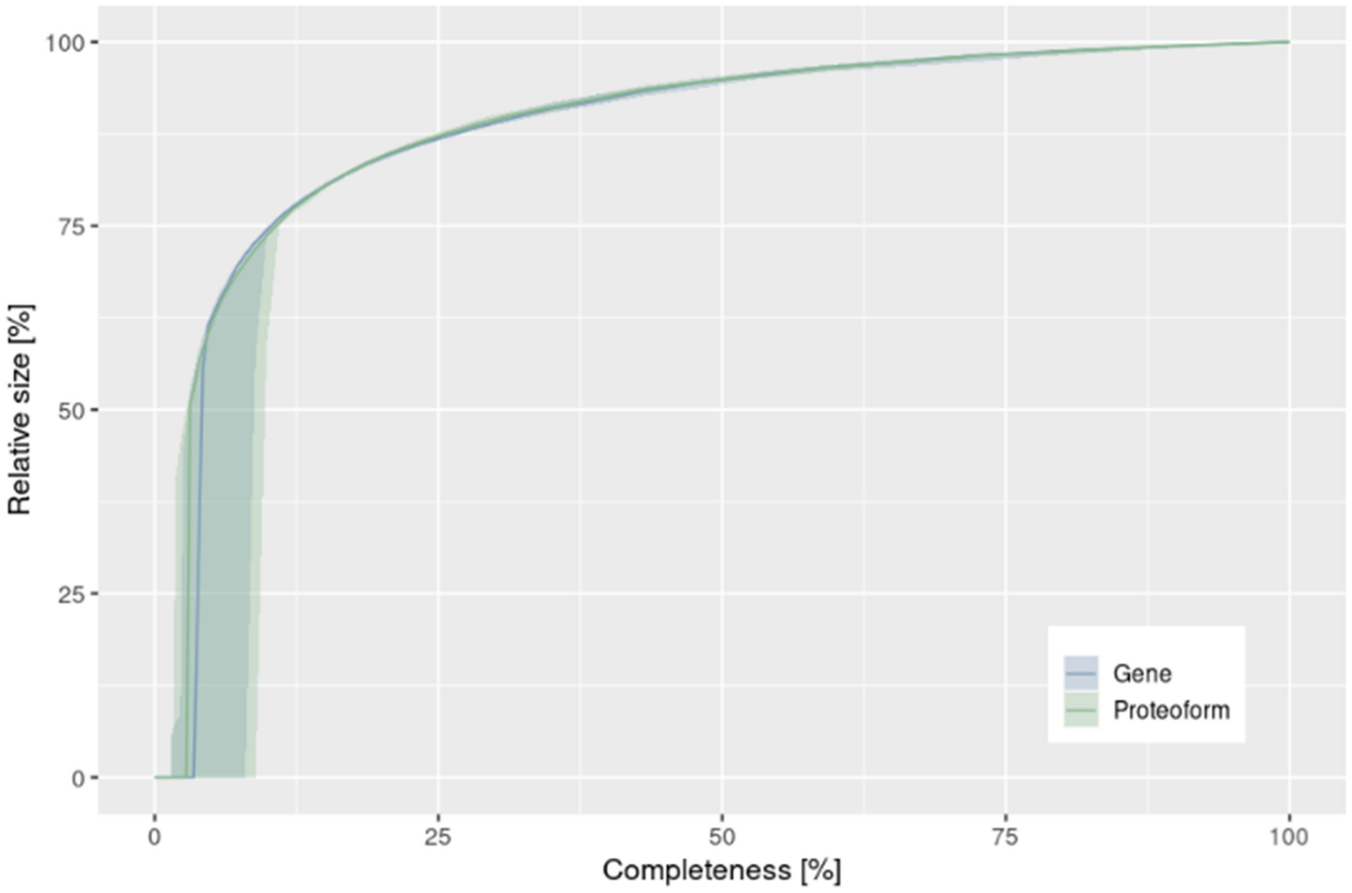
Link percolation curves for gene and proteoform interactome networks. Having the Largest Connected Component (LCC) of a network as the component with the highest number of nodes, the relative size of the LCC (*y* axis) is the number of nodes relative to the number of nodes at start of percolation. Starting with a completeness of 1.0 (complete interactome) and iteratively removing connections until completeness is 0, 20 iterations of percolation were conducted for the gene and proteoform networks. At each completeness level, the solid line shows the median relative size among all iterations and the ribbon the distance between 20th and 80th percentile.

We further evaluated whether the robustness of the network was altered by introducing proteoforms using a percolation analysis. Given that our representation of interaction networks represents an incomplete picture of the interactome, percolation analysis has been suggested to investigate whether a network is dense enough for the systematic analysis of gene sets (Menche *et al*. 2015). Both gene- and proteoform-level interactomes showed similar percolation curves, with very similar curves overlapping until the collapse of the network where the proteoform displays a slightly better robustness, which we ascribe to the cluster of tightly interconnected components visible in Fig. 1B (Fig. 3).

### 3.4 Small molecules reduce the prevalence of isolated components and nodes

**Figure 4.**
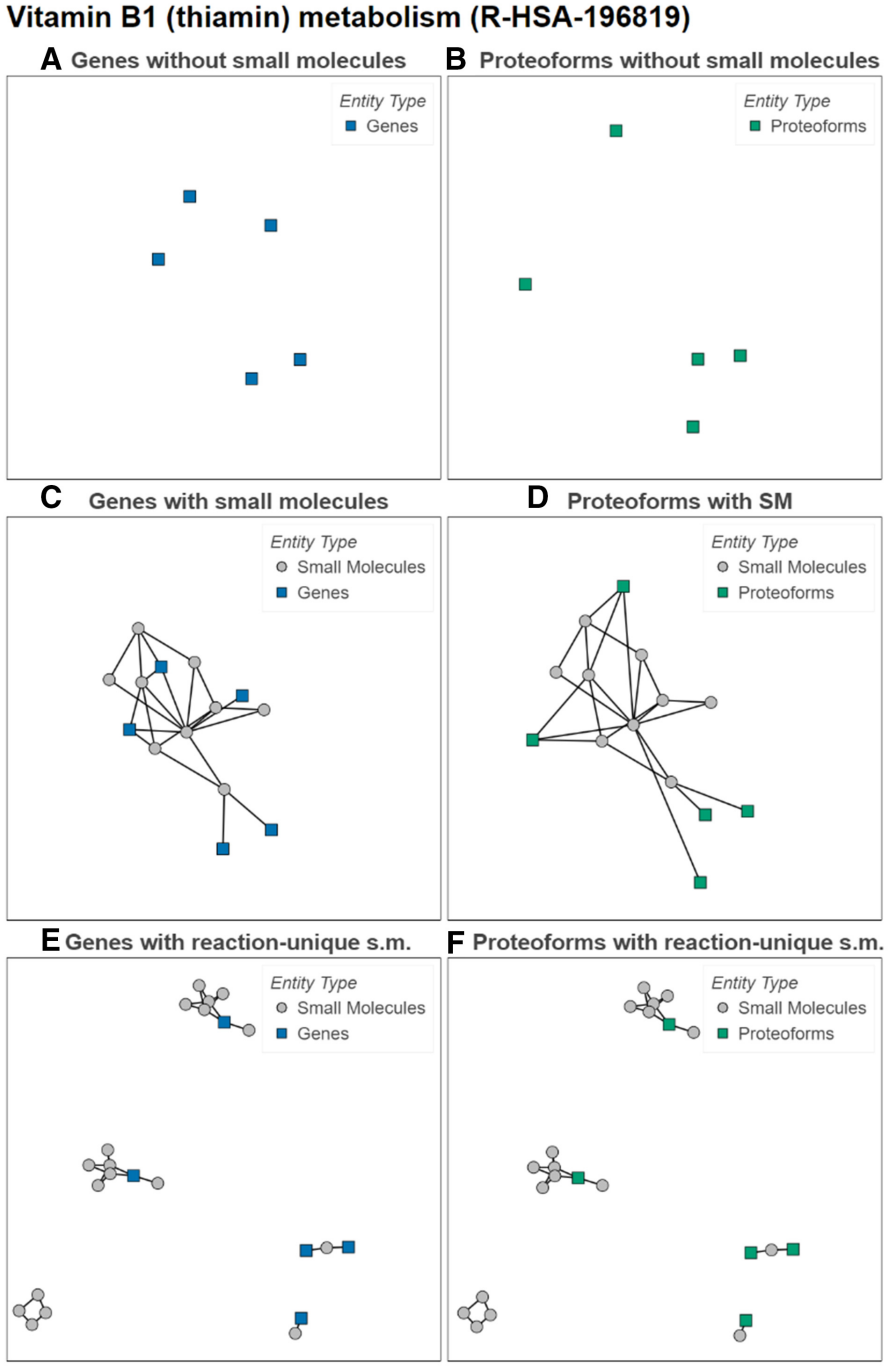
Network representations of Reactome Pathway “Vitamin B1 (thiamin) metabolism” (R-HSA-196819). Squares represent accessioned entities. Gray circles are small molecules. (A) shows how the network consists of only isolated nodes. (B) shows that nodes stay disconnected when using proteoforms. (C) and (D) show how small molecules enable connecting the nodes. (E) and (F) show how reaction-unique small molecules connect isolated nodes by reaction. (SM/s.m.: small molecules).

Adding nodes representing small molecules considerably increases the percentage of nodes part of the LCC, from 85% to 98% in proteoform interactomes. Conversely, adding reaction-unique nodes for small molecules, rather than once for the whole interactome, prevents merging connected components when small molecules are the only nodes shared between reactions. By design, the number of connected components using reaction-unique small molecules is then greater than or equal to the number of connected components obtained when using small molecules, as displayed in Supplementary Table S5, and consequently the LCCs are smaller. At the other end of the scale, some pathways contain proteins performing multiple roles in a reaction but not connected to other proteins, leading to isolated nodes only connected to themselves in the network. This may happen when different isoforms or proteoforms of the same protein participate in the reaction with different roles, resulting in the gene centric representation being a single node interacting with itself while the proteoform-centric representation would show a subnetwork composed of multiple nodes. We found 1665 and 1696 isolated nodes for the gene- and proteoform-centric networks, respectively, showing an overall stable number of isolated nodes. For example, the reactions sustaining Vitamin B1 (thiamin) metabolism (Fig. 4) yield isolated nodes that stay isolated even in the proteoform-centric representation.

Adding small molecules reduces the number of isolated nodes to 164 and 174, respectively. Among the 11 039 accessioned entity nodes, 2715 (24.5%) are connected only through small molecules. When considering 1134 pathways, 226 displayed less isolated nodes when considering small molecules. Conversely, for 40 pathways there were more isolated nodes when adding small molecules. When the studied network is sparse or with many disconnected nodes, it thus becomes useful to include small molecules. They show indirect ways to reach one node from another through reactions, yet the relevance of connecting two distant entities by a small molecule can be questioned. Reaction-unique small molecules provide a balance between reducing the number of isolated nodes while not connecting nodes across different pathways. They allow connecting otherwise isolated nodes through a path that alternates between accessioned entities and reaction-relevant small molecules, while preserving the disconnection of pathways and components.

### 3.5 Articulation points and bridges

Articulation points and bridges are respectively nodes and connections that, if removed, disconnect a connected component into two or more components (Fig. 2). They are thus essential members of the network, maintaining the connection between otherwise disconnected subnetworks. A network is considered more robust when it contains fewer articulation points and bridges. We investigated whether the prevalence of bridges and articulation points changed from a gene-centric to a proteoform-centric representation. However, as detailed in Supplementary Tables S7 and S8 for articulation points and bridges, respectively, adding proteoform annotation does not substantially change the share of articulation points (from 2.45% to 2.5% of nodes). Articulation points in the gene-centric network either stay articulation points in the proteoform-centric network or become more connected due to the multiplicity of proteoform nodes in a proteoform family. Therefore, the use of proteoform nodes creates more connected components rather than creating isolated nodes (Supplementary Table S5). This indicates that, although proteoform annotation increases the connectivity in the network, it is mainly through within-component connectivity.

Given the ubiquitous nature of some small molecules, which participate in many pathways across many contexts, it can be anticipated that they create new connections between connected components. Indeed, adding small molecules reduced the prevalence of bridges and articulation points. In proteoform-centric networks they reduce from 356 (2.46%) to 257 (1.57%). In the network extended with small molecules, 40% of articulation points were small molecules and 60% accessioned entities (Supplementary Table S7). Conversely, when adding reaction-unique small molecules, the number of bridges was tripled, and the robustness of the network hence reduced. Adding reaction-specific small molecules also has the effect of increasing the percentage of proteoforms that are articulation points and increasing the percentage of bridges going out of small molecule nodes, from 3.57% to 10.78% (Supplementary Table S8).

We investigated the changes in prevalence and nature of bridges and articulation points at the level of pathways. Supplementary Tables S9 and S10 detail the averaged values among all pathways in Reactome considered individually. The share of articulation points considering pathways one by one is slightly higher than when considering the complete interactome, highlighting how interactomes aggregate pathways, overlapping the connections of nodes in different contexts. Once again, accessioned entities are more often articulation points than small molecules, demonstrating their key role in biological processes. Nevertheless, small molecules still represent one third of articulation points. Even per pathway, the tendency of small molecules to reduce the percentage of bridge connections is clear, confirming the important role of small molecules for the connectivity of the network at both local and global levels.

Bridges connect more than twice as often accessioned entities rather than small molecules, and the prevalence increases when studying per pathway than for the complete interactome. This increase can be interpreted as the connections of proteoforms conveying more unique information, whereas small molecules may connect more diverse types of other molecules. Reaction-unique small molecules are expected to be articulation points more frequently than regular small molecules, but no difference was found on average. Reaction-unique small molecules increase the total number of articulation points by increasing the percentage of accessioned entity nodes that are articulation points. This is due to the smaller average node degree of the reaction-unique small molecules, compared to the regular small molecules. Hence, when they connect to an accessioned entity, they may convert that accessioned entity into an articulation point.

## 4 Discussion

This study investigated the impact of changing the network representation through the inclusion of proteoforms and small molecules. We based these findings on the Reactome knowledgebase, which contains rich information on biological pathways. Due to the high level of detail on biochemical reactions required to build such networks, functional annotation on proteoforms and small molecules is still scarce. The rapid pace in increase of functional knowledge indicates that such analyses will become increasingly powerful. As the interactome becomes more connected, refining its representation using the rich information available in pathway knowledgebases represents a promising avenue to tease apart densely connected functional regions. Our analysis focused on proteoforms and small molecules, but other information can be included to refine network representations including the directionality of interaction (from input to output), conditioning on previous reactions, participation in complexes or sets, subcellular or biological location.

By modeling the incomplete interactome Menche *et al.* (2015) identified disease modules, and studied their topological properties and pairwise relationships. An overlap between disease modules would then indicate a functional relationship, hinting at shared mechanisms and possible common drug targets. We hypothesize that conducting such analyses with more detailed networks could change the topology at the interface of disease modules, e.g. the connectivity of some modules might decrease when considering proteoform specificity. Furthermore, having an indication that disease modules share, or are connected through, proteoform-specific events, e.g. a phosphorylation, could influence considerations on druggability.

Factors such as analytical challenges, research interest, and literature curation lead to some proteins or pathways to be better annotated than others. The better annotated pathways give a more detailed representation of the biological processes, while understudied proteins or pathways have a much less mature representation or even remain undiscovered. Such biases have a strong influence on the representation of the biological processes involved, and dramatically alter the ability to conduct refined studies such as proteoform-level network analyses. The disparity in biological functional knowledge is a strong limitation of the field, yielding to a network where some processes yield densely connected subnetworks of proteoforms and small molecules, while others are only represented by sparse disconnected gene names—when any information is available at all.

Technologies to identify proteoforms and small molecules are improving constantly but integrating these biological entities in pathways at scale poses numerous challenges. It is therefore important to develop new biological network analysis approaches that can handle the heterogeneity in pathway annotation without losing the rich information gathered by the scientific community. One can envision that such approaches will be generalizable to hybrid networks combining pathway knowledgebases with interaction networks derived from experiments or text mining.

Constructing an interaction network using refined information like proteoforms or small molecules is even more challenging using multiple sources of data. Functional annotations often refer only to gene or protein accessions (Luck *et al.* 2020), hence overlooking post-translational regulatory mechanisms central to many biological processes. The broad adoption of proteoforms in the representation of biological processes is essential to generalize the approaches presented in this study, and hence allow the refinement of the representation of biological processes, which will eventually provide biomedical researchers with more powerful tools to interpret their data.

## Supplementary Material

btad598_Supplementary_Data
