# Supplementary material for: Extending protein interaction networks using proteoforms and small molecules
Source: Bioinformatics. 2023 Sep 26;39(10):btad598. doi: 10.1093/bioinformatics/btad598 (PMC10564616; doi:10.1093/bioinformatics/btad598)
Supplement: btad598_Supplementary_Data [file btad598_supplementary_data.zip › Supplementary_figures_23.06.02.pdf]

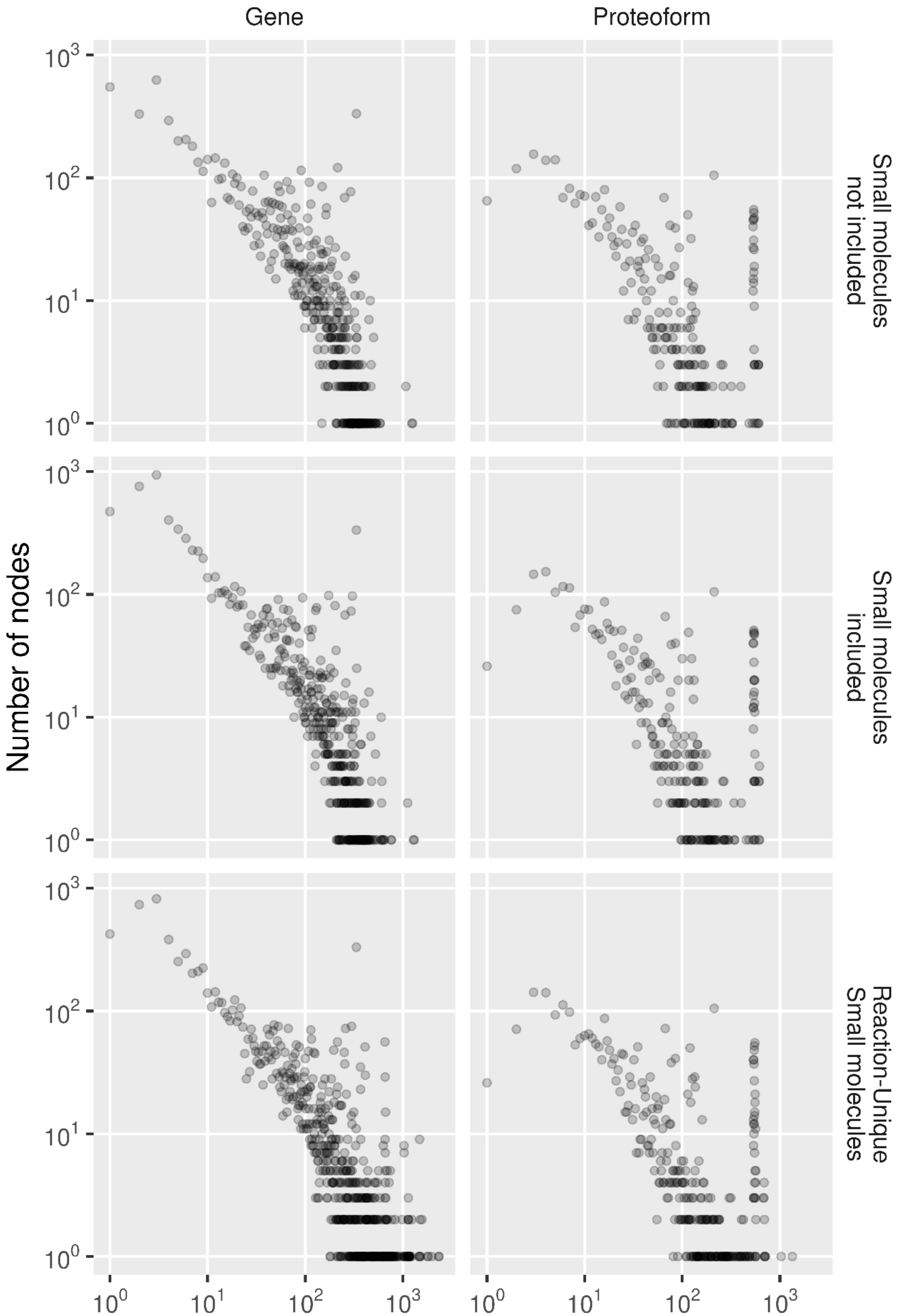

Gene
Proteoform
Small molecules not included
Small molecules included
Reaction-Unique Small molecules
Number of nodes
Degree
10^0
10^1
10^2
10^3

Supplementary Figure 1: Node degree distribution for nodes with isoform or modification information depending on how small molecules are considered and whether proteoforms are considered as individual nodes or collapsed per gene. Each point represents the number of nodes with a given degree plotted against the degree (base 10 logarithmic scale).

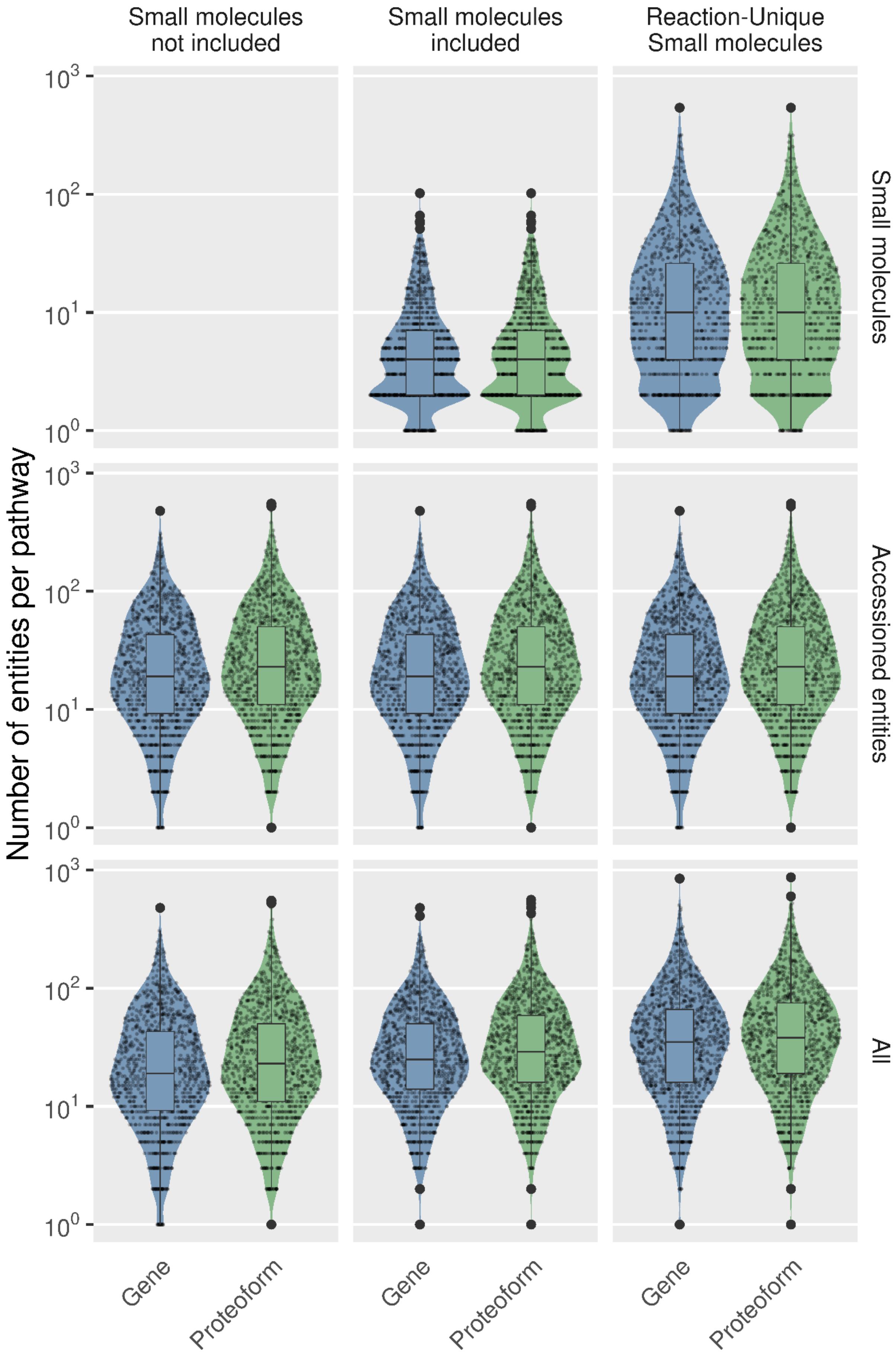

Small molecules not included
Small molecules included
Reaction-Unique Small molecules
Small molecules
Accessioned entities
All
Number of entities per pathway
10^0
10^1
10^2
10^3
Gene
Proteoform

Supplementary Figure 2: Violin, sina, and box plot of the number of participants per pathway subnetwork in the gene- and proteoform-centric interactomes depending on how small molecules are considered. Plots showing the number of small molecules, accessioned entities, and the sum of both are plotted on different rows. Each point represents the number of entities in a given pathway (base 10 logarithmic scale).
